# Recombinant human growth hormone and brain neoplasm association: a pharmacovigilance and Mendelian randomization analysis based on US FAERS, Japanese JADER, and Canadian CVARD

**DOI:** 10.3389/fphar.2025.1630843

**Published:** 2025-07-29

**Authors:** Li Huang, Mei Zhang, Fang Li, Yinpeng Xu

**Affiliations:** ^1^ Department of Pharmacy, Women and Infants Hospital of Zhengzhou, Zhengzhou, Henan, China; ^2^ Department of Pharmacy, The Ninth People’s Hospital of Zhengzhou, Zhengzhou, Henan, China

**Keywords:** recombinant human growth hormone(rhGH), brain neoplasm, FAERS, JADER, CVARD, Mendelian randomization analysis(MR)

## Abstract

**Objective:**

This study aimed to analyze the statistical association between recombinant human growth hormone (rhGH) and brain neoplasm adverse events (AEs) by mining data from the US Food and Drug Administration Adverse Event Reporting System (FAERS) database, the Japanese Adverse Drug Event Report (JADER) database, and the Canada Vigilance Adverse Reaction Online Database (CVARD). Furthermore, Mendelian randomization (MR) was utilized to evaluate the potential causal link between rhGH and brain neoplasm, thereby providing a reference for safe clinical medication use.

**Methods:**

Reports of brain neoplasm associated with rhGH originated from the FAERS database (Q1 2004 - Q4 2024), the JADER database (April 2004 - December 2024), and the CVARD database (January 1991 - December 2024). Disproportionality analysis methods, including the Reporting Odds Ratio (ROR), Proportional Reporting Ratio (PRR), and Information Component (IC), were used to detect pharmacovigilance signals. Subsequently, a two-sample Mendelian randomization analysis was conducted, treating rhGH as the exposure and brain neoplasm as the outcome, to evaluate the causal relationship.

**Results:**

A total of 323 reports of rhGH-associated brain neoplasms AEs were identified (FAERS: n = 282; JADER: n = 14; CVARD: n = 27). Brain neoplasm was detected as a positive signal in all three databases by all three signal detection methods, consistently classified as medium clinical priority. Multivariable logistic regression analysis demonstrated an independent association between age and the increased risk of rhGH-induced brain tumors. Specifically, age groups 18–45 years and 46–65 years showed significantly elevated risks [OR (95% CI): 3.71 (1.09–13.34), *P* = 0.048; and 3.78 (1.02–14.62), *P* = 0.049, respectively]. The two-sample MR analysis, using the Inverse Variance Weighted (IVW) method, yielded an OR of 1.415 (95% CI: 1.005, 1.991, *P* = 0.046), suggestive of causal relationship between rhGH and brain neoplasms.

**Conclusion:**

Pharmacovigilance analysis revealed a significant statistical association between rhGH and brain neoplasms. Mendelian randomization analysis further suggested a causal relationship between them. These findings highlight the need to consider the potential risk of brain neoplasm adverse events during treatment with rhGH.

## 1 Introduction

Recombinant human growth hormone (rhGH), since its approval by the U.S. Food and Drug Administration (FDA) in 1985, has become a key biologic agent for treating conditions such as idiopathic short stature (ISS) and growth hormone deficiency (GHD). Globally, as the prescription volume of rhGH continues to grow, its potential association with the occurrence of brain neoplasms has garnered significant attention. Brain neoplasms are common diseases of the central nervous system, with an incidence rate of 7.72 per 100,000 population and a mortality rate of 4.10 per 100,000 population, representing a major threat to human health. Previous studies regarding the relationship between rhGH and brain neoplasms have yielded inconsistent conclusions. For example, a 2019 prospective study by Child et al., involving 22,311 children from 30 countries, reported comparable neoplasm recurrence rates among children with a prior history of cancer, whether they received rhGH treatment (6.1%) or not (6.8%) (Child et al., 2019). However, a study by Niu et al., in 2002 suggested that brain neoplasms rapidly progressed or recurred after the initiation of growth hormone therapy (Niu et al., 2002).

There is strong evidence from experimental data in cellular and murine models for the role of GH in the development and support of abnormal cell growth, suggesting a plausible biological basis for its involvement in cancer initiation or progression (Sävendahl et al., 2020). A literature review of published combined post-marketing registry data from 1988 to 2016 found that among adults receiving daily GH, the most common causes of death were infections, cardiovascular disease, and malignancies (Bolier et al., 2023). In a study involving 50 patients with non-anterior pituitary parasellar tumours treated with GH, four patients experienced an apparent increase in tumour volume (Chung et al., 2005). Data from the PATRO Adults study, which assessed the long-term treatment with biosimilar somatropin, suggest that an increased risk of second new malignancies in patients with previous cancer cannot be excluded based on the current dataset (Beck-Peccoz et al., 2020). Therefore, the safety of clinical rhGH administration warrants continued attention as an important issue requiring ongoing surveillance.

In recent years, researchers worldwide have conducted large-scale statistical and clinical analyses of extensive spontaneous reporting system (SRS) databases for adverse events (AEs), such as the FAERS, JADER and CVARD. These analyses serve not only to assess the safety of drugs in clinical use (Chiappini et al., 2022; Ide et al., 2024; STM and Longo, 2020), but also to facilitate post-marketing surveillance of adverse drug events by regulatory agencies, thereby mitigating potential harm (Meng et al., 2019). Utilizing single nucleotide polymorphisms (SNPs) as instrumental variables (IVs) derived from genome-wide association studies (GWAS), MR allows for the assessment of causal relationships between drug exposure and adverse outcomes. Given the random assortment of genetic variants at conception, analogous to the randomization process in clinical trials, MR analysis is less prone to confounding and reverse causation biases commonly encountered in traditional observational studies, which are often prone to these biases. Therefore, this study integrates pharmacovigilance analysis using data from the FAERS, JADER, and CVARD databases with MR analysis to investigate the association and potential causal link between rhGH use and brain neoplasm adverse events, aiming to provide further evidence to inform the safe clinical application of rhGH.

## 2 Materials and methods

### 2.1 Source and handling of data

Adverse event data related to rhGH and brain neoplasms in this study were reconstructed based on data obtained from the FAERS for the period Q1 2004–Q4 2024,the JADER covering April 2004 to December 2024, and the CVARD from January 1991 to December 2024. Data for the Mendelian randomization analysis were obtained from the GWAS data of the human plasma proteome genome map (Sun et al., 2018) and the GWAS data of the human phenotype genetic association map (Sakaue et al., 2021).

### 2.2 Data processing

Adverse events related to brain neoplasms were classified and described according to the Medical Dictionary for Regulatory Activities (MedDRA) version 27.1, utilizing the System Organ Class (SOC), High Level Group Term (HLGT), High Level Term (HLT), and Preferred Term (PT) levels. The specific PT code 10061019 was identified for brain neoplasms. Before conducting pharmacovigilance analysis, generic drug names were standardized. Duplication among adverse event reports was addressed by examining criteria such as report ID, sex, and country. For the Mendelian randomization analysis, SNPs were identified as instrumental variables from the GWAS database of recombinant human growth hormone. SNPs were initially screened at a significance level of *P* < 5 × 10^−6^ to ensure a strong association with the exposure. Subsequently, SNPs exhibiting linkage disequilibrium (LD) were removed. Finally, the F-statistic was calculated for the remaining SNPs, and only instrumental variables with an F-statistic >10 were selected to mitigate potential bias from weak instruments. All data organization, extraction, analysis, and plotting were performed using R Studio.

### 2.3 Data analysis

#### 2.3.1 Pharmacovigilance disproportionality analysis

Disproportionality analyses were conducted using a standard 2x2 contingency table (Supplementary Material, Supplementary Table S1) to calculate the Reporting Odds Ratio (ROR), Proportional Reporting Ratio (PRR), and the Information Component (IC) for brain neoplasm reports associated with rhGH (van Puijenbroek et al., 2002). A positive signal was flagged based on the following criteria:ROR: Number of brain neoplasm reports (a) ≥ 3 and the lower limit of the 95% Confidence Interval (CI) of the ROR >1. PRR: Number of Brain neoplasm reports (a) ≥ 3, PRR ≥2, and Chi-square (χ2) ≥ 4. IC: The lower limit of the 95% CI of the IC (IC025) > 0 (Lou et al., 2025; Zhang et al., 2022; Shi et al., 2023) (Supplementary Material, Supplementary Tables S2-S4). In this study, an adverse event was considered a positive signal for rhGH-related brain neoplasm only if detected by at least two of the three methods mentioned above.

#### 2.3.2 Prioritization of relevant disproportionality signals

Adverse events associated with rhGH that met the threshold for signal detection in at least one disproportionality analysis were prioritized using a semiquantitative scoring system based on the criteria detailed in Table 1. Clinical relevance was evaluated based on inclusion in the European Medicines Agency Important Medical Event (IME) and Designated Medical Event (DME) lists. DME are defined as rare but potentially drug-induced serious events. The reporting rate was defined as the proportion of reports for the target adverse event compared to all other adverse events. This was categorized using traditional frequencies: very common (≥10%), common (1%–10%), and uncommon (≤1%). The reported mortality rate was defined as the proportion of reports documenting a fatal outcome among all reports for the target adverse event. Adverse events were assigned an overall priority score based on a total score: 0–2 indicated low priority, 3-5 moderate priority, and 6-8 high priority (Cecco et al., 2024).

**TABLE 1:** Disproportionality analysis yielded criteria and relevant scores for prioritizing adverse events.

Criterium	2 points	1 point	0 point
Reporting rate (cases/non-cases)	>10%	1%–10%	0%–1%
Signal stability (consistency across disproportionality analyses)	3 of 3	2 of 3	1 of 3
Reported case fatality rate (proportion of reports with death asoutcome)	>50%	25%–50%	<25%
Clinical relevance (serious likely drug-attributable AEs)	DME	IME	None

#### 2.3.3 Logistic regression analysis

We performed univariable and multivariable logistic regression analyses. The independent variables included age and gender. Based on the World Health Organization (WHO) age classifications and relevant literature (Wang et al., 2025), this study categorized age into four groups: <18 years old, 18–44 years old, 45–65 years old, and >65 years old. Gender was categorized into two groups: female and male. A criterion for statistical significance was set at *P* < 0.05.

#### 2.3.4 Mendelian randomization analysis

The causal relationship between rhGH (exposure) and brain neoplasm (outcome) was investigated using a two-sample MR approach. The primary analysis was conducted using the inverse variance weighted (IVW) method, supported by four additional MR methods. Robustness and stability of the results were evaluated through sensitivity analyses, including tests for horizontal pleiotropy (MR Egger) and heterogeneity (Cochran’s Q), as well as leave-one-out analysis and scatter plots. A schematic diagram of the MR design is provided in Supplementary Material as Supplementary Figure S1.

## 3 Results

### 3.1 Results of pharmacovigilance analysis

#### 3.1.1 Data extraction results

**FIGURE 1:**
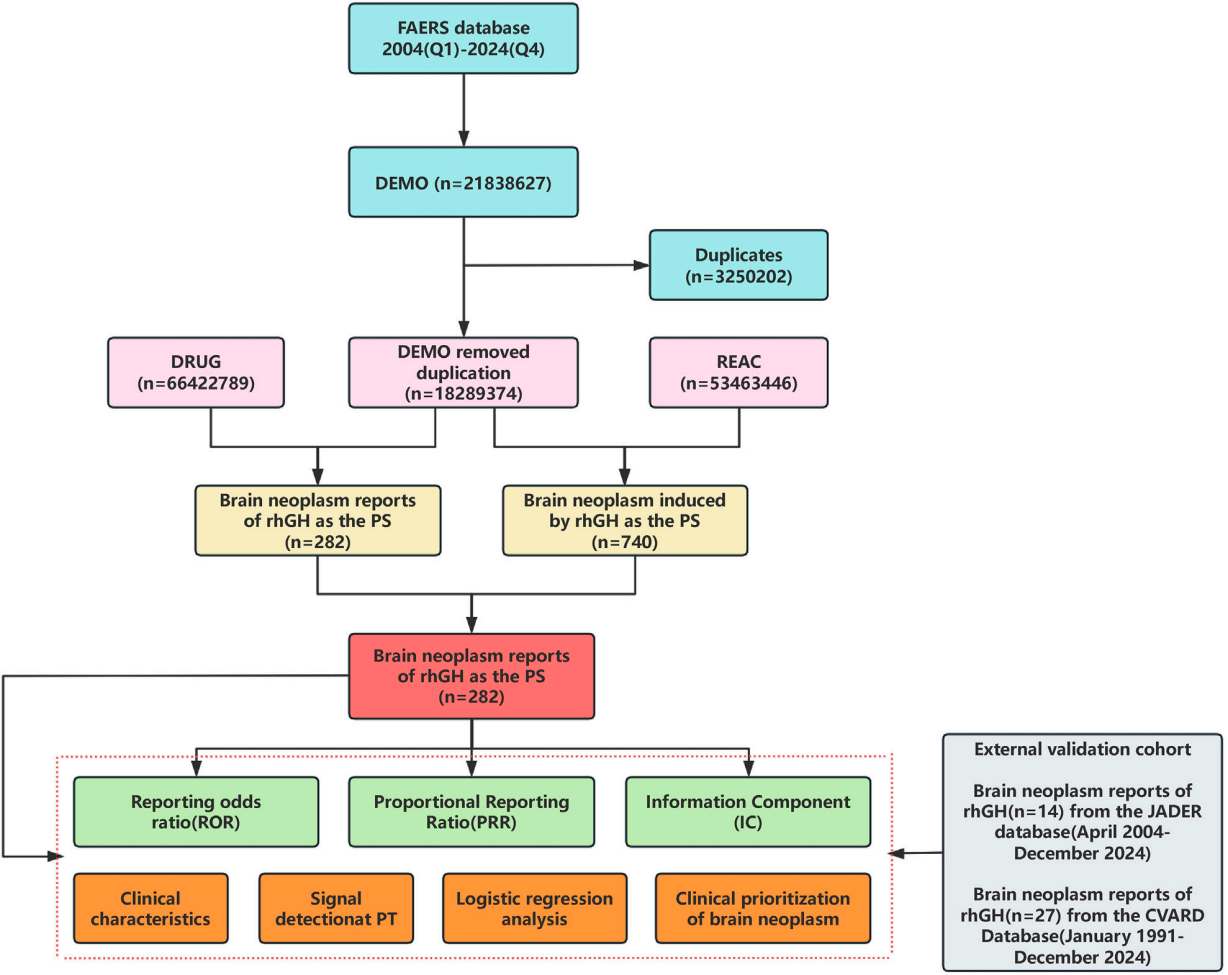
The complete flowchart for the study.

A total of 21,838,627 adverse event reports were received from the FAERS database covering the period from Q1 2004 to Q4 2024. Following data mining and deduplication, 282 reports related to brain neoplasm associated with rhGH were ultimately identified (detailed in Figure 1).

#### 3.1.2 Descriptive overview of cases

**FIGURE 2:**
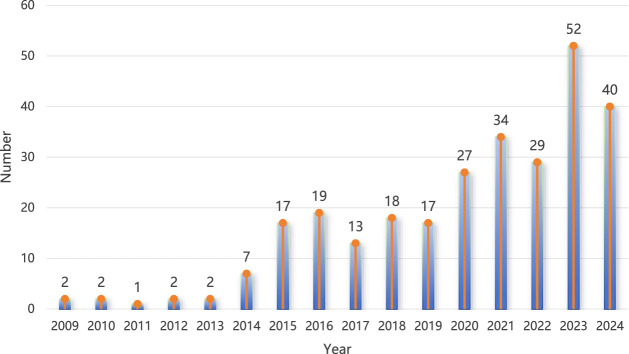
Yearly frequencies of brain neoplasm reports associated with rhGH.

Based on the demographic information of brain neoplasm reports associated with rhGH, the results showed a larger number of male patients (136 cases, 48.2%). The most frequent age group was 35–64 years (63 cases, 22.3%). A considerable proportion of reports indicated hospitalization as an outcome (48 cases, 17.0%). The top five reporting countries were the United States (72 cases, 25.5%), followed by the United Kingdom (71 cases, 25.2%), Canada (42 cases, 14.9%), Colombia (13 cases, 4.6%), and Turkey (11 cases, 3.9%). Detailed demographic statistics are presented in Table 2. Figure 2 illustrates the general trend of the number of reports submitted annually, which showed a general increase over the years.

**TABLE 2:** Characteristics of brain neoplasm reports associated with rhGH from FAERS.

Characteristics	Number of rhGH reports
N [Proportion (%)]	n = 282 (100%)
Gender	
Female	136 (48.2%)
Male	132 (46.8%)
Unknown	14 (5.0%)
Age (year)	
Median (Min-Max)	33 (6–84)
0–11	28 (9.9%)
12–17	37 (13.1%)
18–34	16 (5.7%)
35–64	63 (22.3%)
65–79	9 (3.3%)
≥80	2 (0.7%)
Unknown	127 (45.0%)
Outcome	
Death	6 (2.1%)
Disability	2 (0.7%)
Hospitalization-initial or prolonged	48 (17.0%)
Life-threatening	6 (2.1%)
Unknown	15 (5.3%)
Other outcome	205 (72.7%)
Reported Countries (top fve)	
United States	72 (25.5%)
United Kingdom	71 (25.2%)
Canada	42 (14.9%)
Colombia	13 (4.6%)
Turkey	11 (3.9%)

#### 3.1.3 Disproportionality analysis

**FIGURE 3:**
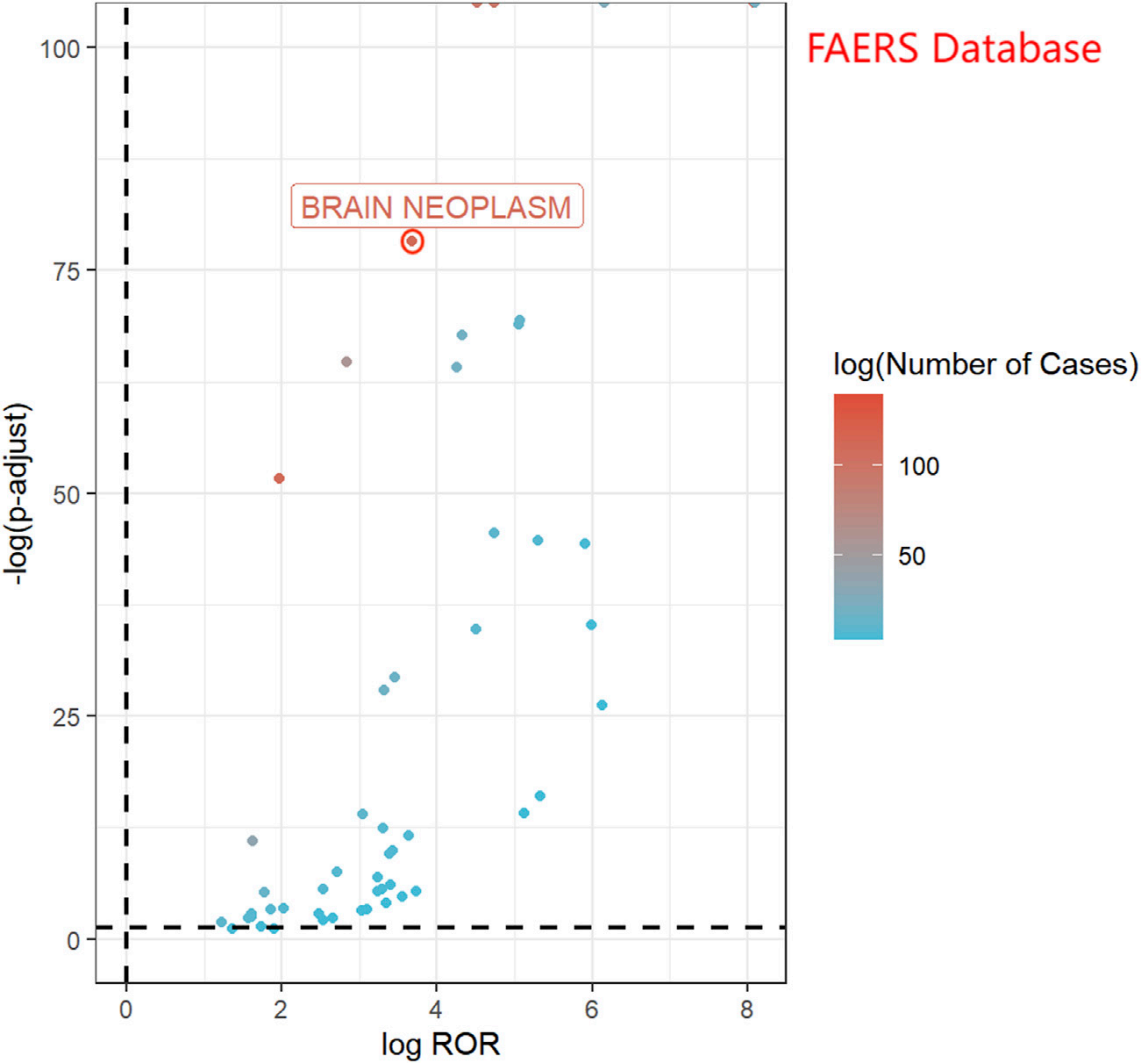
Volcano map of rhGH-associated brain neoplasm adverse reactions logROR from FAERS.

In the FAERS database, 282 reports related to rhGH and brain neoplasms were identified. All three detection methods yielded positive signals (ROR = 12.80, PRR = 12.78, IC = 3.36), as presented in Table 3. Figure 3 displays a volcano plot for the identified rhGH-related adverse events. The y-axis is scaled as−log 10(P−value), representing the p-value obtained from Fisher’s exact test and Bonferroni correction. A value of *P* = 0.05 corresponds to−log 10(P−value)≈1.3; thus larger values on the y-axis indicate a more significant difference. The x-axis is scaled as log (ROR). The color of the points represents the number of case reports, with redder colors indicating a higher number of reports. Therefore, brain neoplasm adverse events located above the dashed line in the fi exhibit significant signal strength and difference.

**TABLE 3:** Detection of brain neoplasm signals associated with rhGH from FAERS.

Drug	PT	Cases(n)	ROR (95% CI)	PRR (χ2)	IC (IC_025_)
rhGH	Brain neoplasm	282	12.80 (11.36–14.41)*	12.78 (2,953.5)*	3.36 (3.45)*

PT, preferred term.

*: Indicates detection of an adverse event risk signal.

#### 3.1.4 Clinical prioritization of relevant disproportionality signals

Table 4 displays the clinical priority analysis of rhGH-related brain neoplasms in the FAERS database, based on four aspects: clinical relevance, reporting rate, fatality rate, and signal stability. The analysis resulted in a total score of 3, classifying it as a moderate priority adverse event. This study confirms the irreplaceable and supplementary role of post-marketing surveillance in detecting rare brain neoplasm adverse events associated with rhGH, thereby supporting proactive clinical monitoring by physicians and promoting safer, more rational drug use.

**TABLE 4:** Clinical priority analysis of rhGH-Related brain neoplasm from FAERS.

PT	Clinical relevance	Reporting rate	Fatality rate (%)	Signal stability	Total score	Relevant prioritization
Brain neoplasm	IME	0.002	0.021	3 of 3	3	moderate priority

IME, important medical event.

#### 3.1.5 Logistic regression analysis

Findings from the univariate logistic regression analysis indicated that sex was not an influencing factor for brain neoplasms associated with rhGH, with no statistically significant difference (*P* > 0.05). Age was significantly associated with brain neoplasm occurrence, with a statistically significant difference at the *P* < 0.05 level. To further investigate the associated risk factors for brain neoplasms, multivariate logistic regression analysis was conducted, demonstrated an independent association between age and the increased risk of rhGH-induced brain tumors. Specifically, age groups 18–45 years and 46–65 years showed significantly elevated risks [OR (95% CI): 3.71 (1.09–13.34), *P* = 0.048; and 3.78 (1.02–14.62), *P* = 0.049, respectively]. Table 5 presents detailed results.

**TABLE 5:** The univariable logistic regression and multivariable logistic regression analysis of factors influencing the brain neoplasm associated with rhGH.

Factors	Univariable			Multivariable		
	OR	95% CI	P	OR	95% CI	P
Age (years)						
<18	1.00 (References)	-	-	-	-	-
18–45	4.07	1.30–10.82	p = 0.008*	3.71	1.09–13.34	p = 0.048*
46–65	4.18	1.47–10.61	p = 0.004*	3.78	1.02–14.62	p = 0.049*
>65	2.28	0.36–8.30	p = 0.278			
Gender						
Female	1.00 (References)	-	-	-	-	-
Male	0.66	0.30–1.45	p = 0.297	-	-	-

The exposure factors considered included gender and age.

OR, indicates odds ratio.

* indicates significant, P < 0.05.

#### 3.1.6 External validation in JADER/CVARD database

**FIGURE 4:**
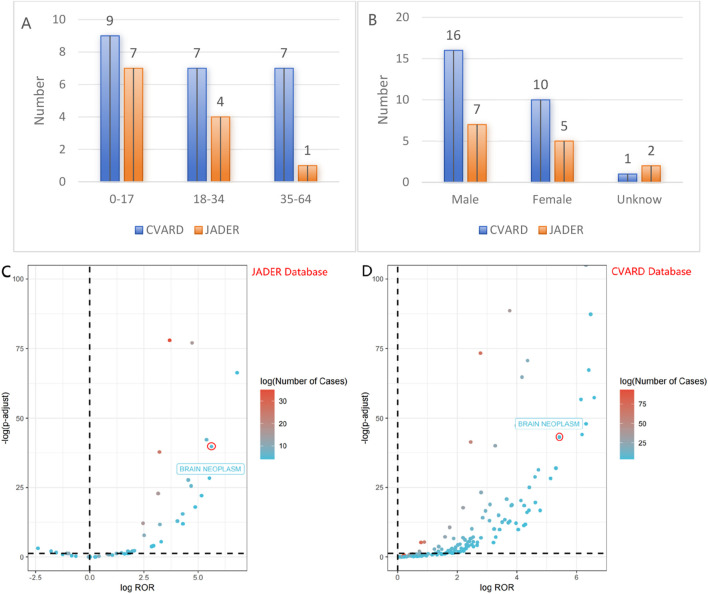
The fi present statistical information on age and sex of reported patients **(A,B)**, volcano map of rhGH-associated brain neoplasm logROR from JADER/CVARD **(C,D)**.

To validate the findings from the FAERS, data from the JADER and CVARD databases were utilized. A total of 14 reports of rhGH-related brain neoplasm was collected from the JADER from April 2004 to December 2024, and 27 reports were collected from the CVARD from January 1991 to December 2024. In both databases, all three detection methods indicated a positive signal for rhGH-related brain neoplasms (JADER database: ROR = 87.13, PRR = 86.25, IC = 6.32; CVARD database: ROR = 45.88, PRR = 45.59, IC = 5.48), as shown in Table 6. Furthermore, the clinical priority of rhGH-related neoplasms in both databases was consistently classified as moderate priority (Table 7). Demographic statistics regarding the age and sex of reported patients are presented in Figures 4A,B. Figures 4C,D displays volcano plots for rhGH adverse events in the JADER and CVARD databases. The signals for rhGH-related brain neoplasm identified in the JADER and CVARD databases were consistent with those found in the FAERS database.

**TABLE 6:** Detection of brain neoplasm signals associated with rhGH from JADER/CVARD.

Database	PT	Cases(n)	ROR (95% CI)	PRR (χ2)	IC (IC_025_)
CVARD	Brain neoplasm	14	87.13 (50.45–150.46)*	86.25 (1,095.1)*	6.32 (4.64)*
JADER	Brain neoplasm	27	45.88 (26.97–78.06)*	45.59 (597.03)*	5.48 (3.81)*

PT, preferred term.

*: Indicates detection of an adverse event risk signal.

**TABLE 7:** Clinical priority analysis of rhGH-Related brain neoplasm from JADER/CVARD.

Database	PT	Clinical relevance	Reporting rate	Fatality rate (%)	Signal stability	Total score	Relevant prioritization
JADER	Brain neoplasm	IME	0.010	0	3 of 3	4	moderate priority
CVARD	Brain neoplasm	IME	0.007	0	3 of 3	3	moderate priority

PT, preferred term; IME, important medical even.

### 3.2 Results of Mendelian randomisation analysis

We performed a two-sample MR analysis to examine whether rhGH is causally related to brain neoplasm. The specific analysis was performed as follows.

#### 3.2.1 Data sources and instrumental variable selection

Genetic data for rhGH (exposure) were obtained from the human plasma proteome genome map GWAS data published by Sun et al., in 2018, which included 3,301 participants (Sun et al., 2018). Genetic data for brain neoplasm (outcome) were obtained from the human phenotype genetic association map GWAS data published by Sakaue et al., in 2021, which included 178,726 participants (Sakaue et al., 2021). Instrumental variables were selected as genome-wide significant SNPs (*P* < 5 × 10^−6^). To ensure the strength of the instrumental variables and avoid linkage disequilibrium (LD), selected SNPs were filtered to have an r^2^ value less than 0.001 within a 10,000 kb window. Furthermore, to avoid weak instrument bias, instrumental variables with an F-statistic >10 were selected. Bone morphogenetic protein 6 (BMP6) was included as a positive control; its genetic data were also obtained from the human plasma proteome genome map GWAS data by Sun et al., (2018), including 3,301 participants (Sun et al., 2018). Specific data details are presented in Supplementary Material as Supplementary Table S5.

After removing linkage disequilibrium among the instrumental variables, 23 significant SNPs were selected. The F-statistic values ranged from 20.85 to 25.33, and all were >10, indicating the absence of weak instrumental variables. Basic information about these specific SNPs is presented in Table 8.

**TABLE 8:** Basic information of SNPs associated with rhGH.

Substance	SNPs	CHR	POS	EA	OA	EAF	Β	SE	*P.* value	F
rhGH	rs113740320	1	29179467	T	G	0.06699	0.2367	0.0497	1.90e-06	22.66
	rs149451734	2	3955107	A	G	0.02162	−0.4357	0.0944	3.89e-06	21.28
	rs17762054	2	163010686	C	T	0.02211	0.3993	0.0843	2.18e-06	22.42
	rs10004661	4	182498876	A	C	0.34907	−0.1198	0.0259	3.63e-06	21.38
	rs1380012	4	179052399	C	T	0.75385	0.1407	0.029	1.23e-06	23.52
	rs7757428	6	91502332	G	A	0.32895	0.1216	0.0263	3.63e-06	21.36
	rs1044043	6	32793981	C	A	0.78396	−0.137	0.0299	4.67e-06	20.98
	rs62521900	8	141901195	T	G	0.01852	−0.4771	0.1016	2.63e-06	22.03
	rs13252141	8	11128104	T	C	0.19011	0.1593	0.0323	7.76e-07	24.30
	rs7873013	9	122676273	T	G	0.6277	0.1299	0.0258	4.67e-07	25.33
	rs7142713	14	28313517	A	G	0.27272	−0.1313	0.0281	2.95e-06	21.81
	rs3742848	14	71191423	T	C	0.5992	−0.1205	0.0252	1.69e-06	22.85
	rs73469695	15	98631091	C	T	0.0481	0.2749	0.0589	3.01e-06	21.76
	rs145701180	16	31725945	G	A	0.02487	0.4228	0.0896	2.34e-06	22.25
	rs58124292	16	81890435	A	G	0.20348	−0.1517	0.0318	1.81e-06	22.74
	rs59902361	20	38334734	A	G	0.02218	−0.391	0.0856	4.89e-06	20.85

#### 3.2.2 Positive control analysis

**FIGURE 5:**
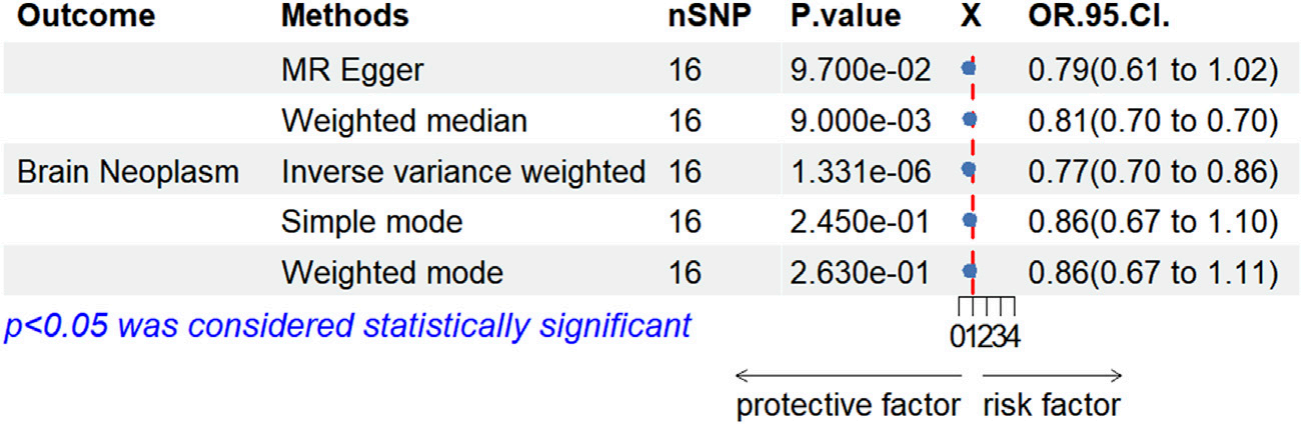
Forest image of MR Analysis of bone morphogenetic protein 6 by rhGH.

We conducted a two-sample MR analysis between rhGH and bone morphogenetic protein 6. The IVW analysis yielded OR of 0.77 (95% CI: 0.70–0.86, *P* = 1.331 × 10^−9^), indicating that rhGH acts as a protective factor for bone morphogenetic protein 6. The direction of this association is consistent with clinical results, which suggests the validity of the instrumental variables. Specific results are presented in Figure 5.

#### 3.2.3 Mendelian randomisation analysis of rhGH on brain neoplasm

IVW-MR analysis indicated that rhGH is associated with an increased risk of Brain neoplasm. The OR was 1.415 (95% CI: 1.005, 1.991, *P* = 0.046), suggesting a statistically significant causal effect. Specific results are presented in Table 9.

**TABLE 9:** Results of Mendelian randomization of rhGH for brain neoplasm.

Exposure	Outcome	Methods	SNPs	OR (95%CI)	*P*. value
rhGH	Brain neoplasm	MR Egger	9	1.009 (0.478, 2.128)	0.980
		Weighted median	9	1.287 (0.890, 1.861)	0.178
		Inverse variance weighted	9	1.415 (1.005, 1.991)	0.046
		Simple mode	9	1.666 (0.919, 3.019)	0.130
		Weighted mode	9	1.280 (0.891, 1.838)	0.217

#### 3.2.4 Horizontal pleiotropy and heterogeneity tests

Results from the horizontal pleiotropy test using the MR-Egger method indicated no potential horizontal pleiotropy between rhGH and brain neoplasm (*P* = 0.351). The MR regression intercept was 0.069, which is very close to 0, further supporting the absence of horizontal pleiotropy and indicating that the Egger regression model is highly consistent with the IVW method, thus not biasing the results. In the heterogeneity test, the *P*-values from both the MR-Egger and IVW methods were >0.05, indicating that the included instrumental variables did not exhibit significant heterogeneity. Re-testing using the MR-PRESSO method also found no aberrant instrumental variables. Therefore, none of the 9 selected instrumental variables needed to be removed. Specific results are presented in Table 10.

**TABLE 10:** Test of pleiotropy and heterogeneity of rhGH for brain neoplasm.

Exposure	Outcome	Pleiotropy-test			Heterogene-test					
		MR-Egger			MR-Egger			IVW		
rhGH	Brain neoplasm	Intercept	SE	*P* val	Q	Q_df_	Q_pval_	Q	Q_df_	Q_pval_
		0.069	0.069	0.351	10.460	7	0.163	11.950	8	0.153

#### 3.2.5 Sensitivity analysis

**FIGURE 6:**
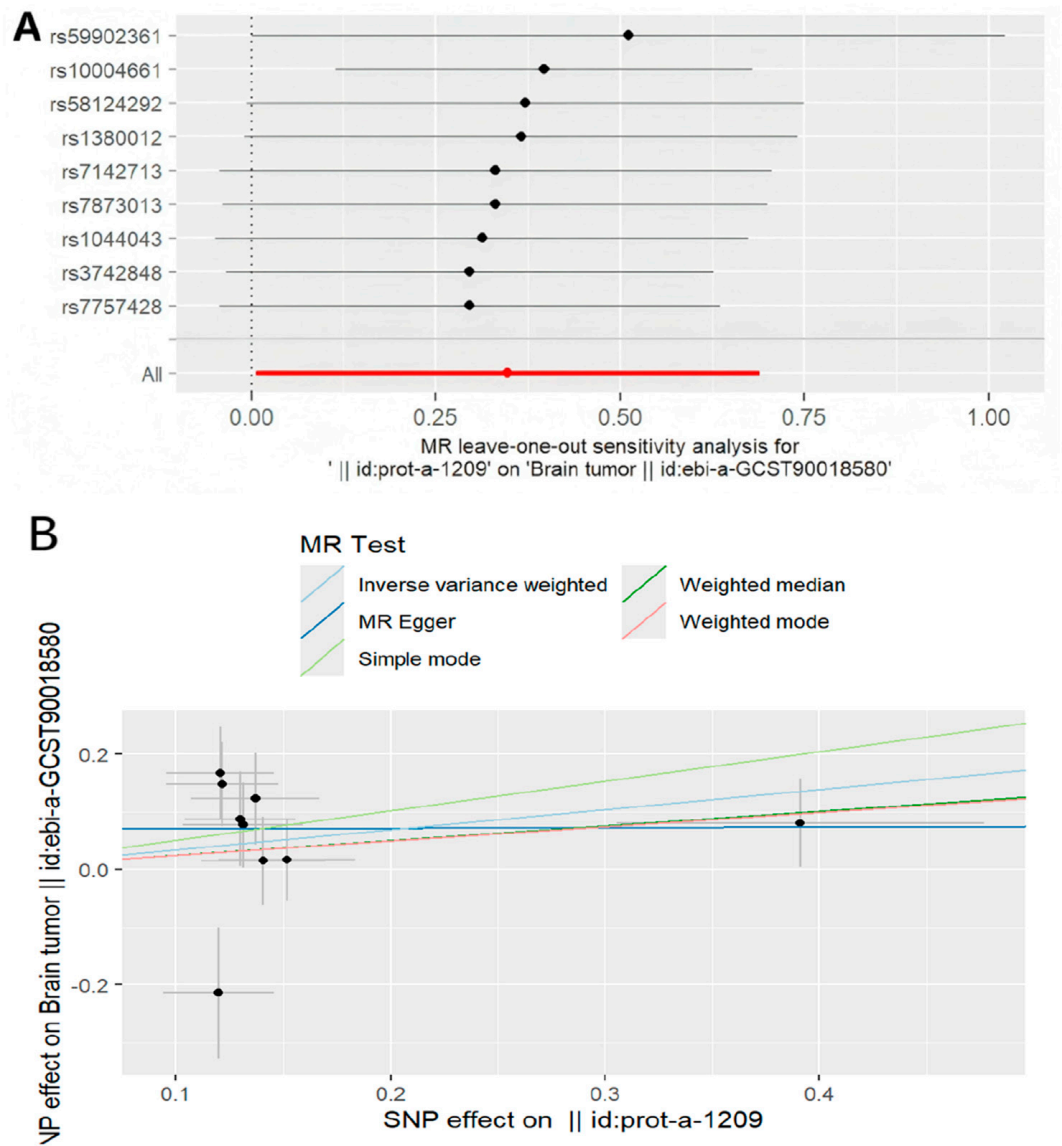
Mendelian randomized association between rhGH and neoplasm **(A)**: Leave-one-out,**(B)**: Scatter diagram.

To evaluate the sensitivity of the MR findings, leave-one-out analysis and scatter plots were utilized, revealed that the overall causal estimate remained robust, as removing any single SNP did not cause a substantial change, suggesting no single nucleotide polymorphism exerted undue influence. This suggests that the study findings are robust (Figure 6A). Scatter plots also showed largely consistent directions of the regression lines for the genetically predicted effect of rhGH on brain neoplasm, suggesting that as exposure to rhGH increases, the risk of brain neoplasm may increase (Figure 6B).

## 4 Discussion

### 4.1 Analysis of results

The objective of this study was to investigate the potential association rhGH and brain neoplasm using a dual approach: pharmacovigilance analysis of real-world adverse event reporting systems and a two-sample MR analysis to explore potential causality. Our findings from the pharmacovigilance analysis across FAERS, JADER, and CVARD databases consistently detected a significant disproportionality signal for brain neoplasm associated with rhGH. This was further supported by MR analysis, which suggested a potential causal relationship between genetically proxied rhGH levels and an increased risk of brain neoplasms.

The pharmacovigilance data, encompassing over 2 decades, revealed 282 reports of brain neoplasms linked to rhGH in the FAERS database alone. The calculated ROR (12.80, 95% CI: 11.36–14.41), PRR (12.78), and IC (3.36) values all indicated a strong signal. This signal was independently corroborated by analyses of the JADER (ROR = 87.13) and CVARD (ROR = 45.88) databases, enhancing the robustness of this observation. The yearly increasing trend in such reports, as seen in Figure 2, warrants attention, potentially reflecting increased rhGH usage, heightened reporting awareness, or a genuine rise in incidence. Demographically, while males constituted a slight majority of reports (48.2%), the 35–64 age group represented the largest proportion of affected individuals (22.3%). Notably, multivariable logistic regression analysis demonstrated an independent association between age and the increased risk of rhGH-induced brain tumors. Specifically, age groups 18–45 years old and 46–65 years old showed significantly elevated risks [OR (95% CI): 3.71 (1.09–13.34), *P* = 0.048; and 3.78 (1.02–14.62), *P* = 0.049, respectively] The clinical priority analysis in all three databases consistently categorized rhGH-associated brain neoplasm as “moderate priority,” underscoring the need for continued surveillance and clinical vigilance.

To further explore the nature of this association and mitigate potential confounding inherent in observational data, we employed a two-sample MR approach. The successful validation of our instrumental variables using bone morphogenetic protein 6 as a positive control (OR 0.77, 95% CI: 0.70–0.86, *P* = 1.331e-9 for rhGH on BMP6), consistent with known biological relationships, lent confidence to our MR methodology. Evidence for a statistically significant association between genetically predicted rhGH levels and an increased risk of brain neoplasms was provided by the primary IVW MR analysis (OR 1.415, 95% CI: 1.005–1.991, *P* = 0.046). Consistent with this finding, other MR methods (MR Egger, Weighted Median, Simple Mode, Weighted Mode) also showed effect estimates pointing in the same direction; however, their results were not statistically significant and presented with wider confidence intervals. This may be due to the lower statistical power of these alternative methods, particularly with the number of SNPs available. Importantly, sensitivity analyses, including MR-Egger regression intercept (*P* = 0.351) and heterogeneity tests (Q-test *P* > 0.05), did not reveal evidence of significant horizontal pleiotropy or heterogeneity that would bias the IVW estimate. The MR-PRESSO analysis also found no outlier IVs, and leave-one-out analysis confirmed that the causal estimate was not driven by any single SNP, suggesting the robustness of the IVW findings.

The convergence of evidence from extensive pharmacovigilance signal detection across multiple international databases and the supportive, albeit modestly significant, causal inference from MR analysis provides a compelling argument for an association between rhGH and brain neoplasm. While the role of GH in normal brain development and function is established, its potential involvement in neoplasia is complex. GH can promote cell proliferation and survival and has been implicated in various cancers, although direct links to brain neoplasm in the general population receiving rhGH have been a subject of ongoing research and debate. Our findings align with concerns that supraphysiological levels or prolonged exposure to rhGH might contribute to oncogenic processes in susceptible individuals. The identified age group of 18–45 years old and 46–65 years old as being at potentially higher risk in the pharmacovigilance data may reflect a period of vulnerability or patterns of rhGH use, which warrants further investigation.

### 4.2 Studies related to rhGH and brain neoplasm

rhGH has been in clinical use for nearly 40 years, and its involvement in stimulating growth and enhancing recovery from severe burns is widely recognized. However, with its increased use, safety concerns, particularly regarding malignancies, have also received increasing attention. Darendeliler conducted post-marketing brain neoplasm surveillance in 50,000 patients treated with rhGH, reporting a neoplasm recurrence rate of 11.7% among brain neoplasm patients (Darendeliler et al., 2006). In 2022, extensive research on various cancer types by Kopchick et al. demonstrated that the intrinsic growth-promoting effect of rhGH can drive the growth and proliferation of cancer cells *in vitro* and *in vivo* (Kopchick et al., 2022). A 2002 study by Swerdlow et al. followed 1848 patients treated with rhGH during childhood/early adulthood (1959–1985), tracking cancer incidence and mortality from December 1995 to December 2000. After controlling for age, sex, and period, they compared cancer risk to the general population and found an approximately 3-fold increase in the overall risk of cancer death (Swerdlow et al., 2002). Chinese research suggests that tumor risk associated with rhGH treatment may be related to high IGF-1 and high GH levels. IGF-1 and GH have mitogenic and anti-apoptotic effects and, in addition to their proliferative effects on normal tissues, are also involved in the occurrence and development processes of various neoplasms, including creating an environment favorable for neoplasm occurrence and promoting the growth of potential neoplasms in the body (Subspecialty Group of Endocrinology, Genetics, and Metabolism, 2013; Pan and Du, 2024). According to research by Yu et al., rhGH is capable of activating signal transducers and activators of transcription (STAT) proteins. Given that dysregulated STAT activity is fundamentally involved in the pathogenesis and progression of human cancers, this finding suggests a potential mechanism (Yu et al., 1995). Therefore, in clinical practice, to mitigate the risk of neoplasm recurrence, a detailed medical history should be taken for all patients. For patients with a history of tumors, rhGH treatment should be used cautiously, comprehensively considering the degree of tumor malignancy and progression status. If necessary, cranial magnetic resonance imaging and laboratory tumor marker tests may be performed to assess for signs of recurrence.

### 4.3 Limitation

This study combined adverse drug reaction databases and Mendelian randomization, an analysis method that can mitigate the influence of potential confounders on the accuracy of association results (Klarin et al., 2020). However, it may also have limitations: (1) The relatively small sample size of the GWAS for the rhGH, which included only 3,301 individuals. A smaller sample size can lead to imprecise estimates of SNP-exposure effect sizes, in turn potentially reducing the strength of the instrumental variables and increasing the risk of weak instrument bias. Although all 23 SNPs selected for our analysis had F-statistics greater than the conventional threshold of 10 (ranging from 20.85 to 25.33), which substantially mitigates concerns about weak instruments, we recognize that the F > 10 criterion does not completely eliminate the possibility of bias in a small-sample GWAS. Despite this limitation, we proceeded with the MR analysis based on three key considerations. First, we performed a leave-one-out sensitivity analysis to assess the impact of this potential bias on our causal inference. The results confirmed that our conclusion was not unduly influenced by any single SNP. Second, this GWAS is one of the few publicly available genetic tools for investigating this question, and our study was intended as a preliminary exploration using the best available evidence. Third, this study highlights the limitations of the current evidence, thereby sending an important message to the academic community: there is an urgent need for larger-scale rhGH-related GWAS to provide sufficient statistical power for drawing more definitive causal inferences. (2) We retrieved 11 reports from Japan and 42 reports from Canada in the FAERS database, which may overlap with reports in the JADER and CVARD databases, respectively. Due to the differing structures of these databases, direct de-duplication was not possible. Nevertheless, the positive ROR/PRR/IC signals calculated based on the number of reports in each database individually still support the statistical association between the drug and the adverse event. (3) The FAERS and JADER databases became publicly available starting from 2004. Therefore, reports of brain tumors associated with recombinant human growth hormone use before 2004 are missing. (4) Using GWAS data for Mendelian randomization analysis cannot explore any potential non-linear relationships or stratification effects across different ages, health statuses, or sexes, which might contribute to heterogeneity.

## 5 Conclusion

This study provides significant pharmacovigilance evidence and supportive genetic evidence suggesting an association between rhGH administration and an increased risk of brain neoplasm, particularly flagging the 18–45 age group and 46–65 age group from reported data. These findings highlight the irreplaceable role of post-marketing surveillance and the utility of MR in evaluating potential adverse drug reactions. While not definitive proof of causality that would alter current prescribing guidelines without further confirmation, our results underscore the importance of careful benefit-risk assessment, individualized treatment decisions, and continued vigilant monitoring for patients receiving rhGH.

Future research should further delve into mechanistic studies and large-scale prospective cohort investigations. For instance: (1) Mechanistic studies on the potential biological links between recombinant human growth hormone and brain tumors. This would involve exploring specific molecular pathways, cellular processes, or genetic interactions that could underpin this association. (2) Studies focusing on specific patient populations treated with recombinant human growth hormone, such as children and adults with growth hormone deficiency, or individuals with severe burns. (3) Methodological advancements using techniques like multivariable or non-linear Mendelian Randomization (MR) to enhance the study’s impact and translational value. This could involve exploring the independent effects of multiple exposures or investigating potential non-linear relationships.

## Data Availability

The original contributions presented in the study are included in the article/Supplementary Material, further inquiries can be directed to the corresponding author.
