# Supplementary material for: Recombinant human growth hormone and brain neoplasm association: a pharmacovigilance and Mendelian randomization analysis based on US FAERS, Japanese JADER, and Canadian CVARD
Source: Front Pharmacol. 2025 Jul 29;16:1630843. doi: 10.3389/fphar.2025.1630843 (PMC12339429; doi:10.3389/fphar.2025.1630843)
Supplement: Supplementary file 1 [file DataSheet1.zip › The data involved in the research - 2/Supplementary materials--2025.7.9.docx]

**Supplementary materials**

**Title:**

**Recombinant Human Growth Hormone and Brain Neoplasm Association: A Pharmacovigilance and Mendelian Randomization Analysis Based on US FAERS, Japanese JADER, and Canadian CVARD**

Li Huang^1^, Mei Zhang^1^,Fang Li^2^, Yinpeng Xu^2^*

1. Department of Pharmacy, Women and Infants Hospital of Zhengzhou, Zhengzhou, 450000, Henan, China
2. Department of Pharmacy, The Ninth People's Hospital of Zhengzhou, Zhengzhou, 450000, Henan, China

* Corresponding author: Yinpeng Xu

Email:suncz87@hhu.edu.cn

**1.Calculation of rhGH-related brain neoplasm signal**

A standard two-by-two contingency table was used to calculate the Reporting Odds Ratio (ROR), Proportional Reporting Ratio (PRR), and the Information Component (IC) for brain neoplasm reports associated with rhGH.

Table S1. Two-by-two contingency table for measure of disproportionality.

|  | **Number of Brain neoplasm reports** | **Number of other AEs reports** | **Total** |
| --- | --- | --- | --- |
| rhGH | A | B | A+B |
| Other Drugs | C | D | C+D |
| Total | A+C | B+D | A+B+C+D |

Table S2. Formula for the ROR and 95% CI

| **Measure of association** | **Formula** | **Threshold value** |
| --- | --- | --- |
| ROR | $ROR=(A/C)/(B/D)=AD/BC$ | The case count ≥3, lower limit of 95% CI >1 |
|  | $SE(\ln ROR)=$^^ |  |
|  | *^^* |  |

Table S3. Formula for the PRR and χ2

| **Measure of association** | **Formula** | **Threshold value** |
| --- | --- | --- |
| PRR | $PRR=\frac{A/(A+B)}{C/(C+D)}$ | The case count ≥3, PRR ≥2, χ^2^ ≥4 |
|  | $\chi^{2}=\frac{N(AD-BC)^{2}}{(A+B)(C+D)(A+C)(B+D)}$ |  |

Table S4. Formula for the IC

| **Measure of association** | **Formula** | **Threshold value** |
| --- | --- | --- |
| IC | $E(IC)=\log_{2}\frac{\gamma(A+\lambda)}{(N+\gamma)}$ | IC_025_ >0 |
|  | $\gamma=\frac{\lambda(N+\sigma)(N+\eta)}{(A+B+\alpha)(A+C+\beta)}$ |  |
|  | $V(IC)=(\frac{1}{\ln2})^{2}[\frac{N-A+\gamma-\lambda}{(A+\lambda)(1+N+\lambda)}+\frac{N-A-B+\sigma-\alpha}{(A+B+\alpha)(1+N+\sigma)}+\frac{N-A-C+\eta-\beta}{(A+C+\beta)(1+N+\eta)}]$ |  |
|  | $IC=E(IC)\text{，}\alpha=\beta=\lambda=1\text{，}\sigma=\eta=2$ |  |
|  | $IC_{025}=IC-2\sqrt{V(IC)}$ |  |

**2.Schematic diagram of the three core assumptions and design of Mendelian randomization analysis**

The analysis relies on three core assumptions: ① strong association between instrumental variables and the exposure; ② independence of instrumental variables from confounders; and ③ instrumental variables influencing the outcome solely via the exposure.


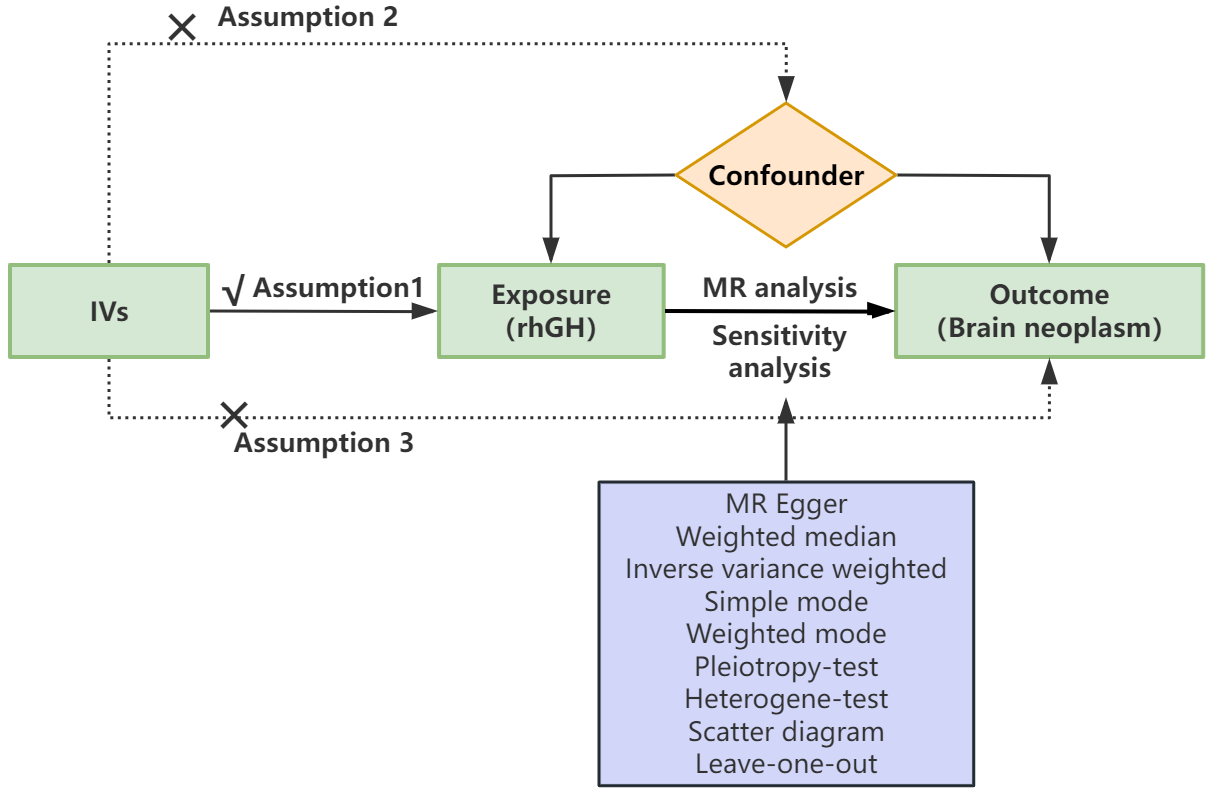


Figure S1. Schematic diagram of Mendelian randomization design

1. **MR Data Sources**

Table S5. Exposure/outcome Sample information

| Exposure / Outcome | Year | Sample size | Number of SNPs | Data sources |
| --- | --- | --- | --- | --- |
| rhGH（Exposure） | 2018 | 3,301 | 10,534,735 | The GWAS data of the human plasma proteome genome atlas studied by Sun et al ^9^. |
| Brain neoplasm（Outcome） | 2021 | 178,726 | 12,454,705 | The GWAS data of human phenotypic genetic association maps studied by Sakaue et al ^10^. |
| Bone morphogenetic protein 6  （Positive control outcome） | 2018 | 3,301 | 10,534,735 | The GWAS data of the human plasma proteome genome atlas studied by Sun et al ^9^. |
