# Supplementary figures and images for: Recombinant human growth hormone and brain neoplasm association: a pharmacovigilance and Mendelian randomization analysis based on US FAERS, Japanese JADER, and Canadian CVARD
Source: Front Pharmacol. 2025 Jul 29;16:1630843. doi: 10.3389/fphar.2025.1630843 (PMC12339429; doi:10.3389/fphar.2025.1630843)

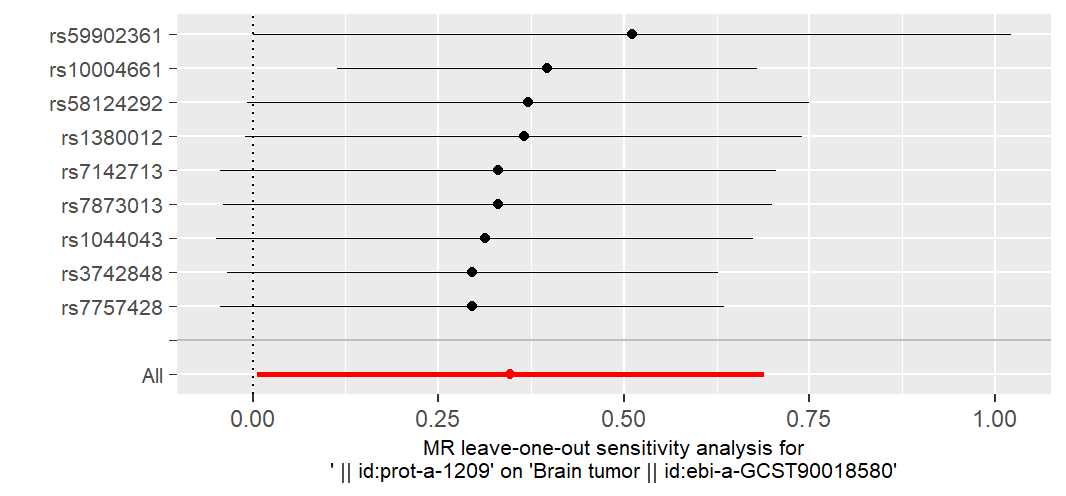

Supplement: Supplementary file 1 [file DataSheet1.zip › The data involved in the research - 2/Mendel Randomization/leave-one-out map.png]

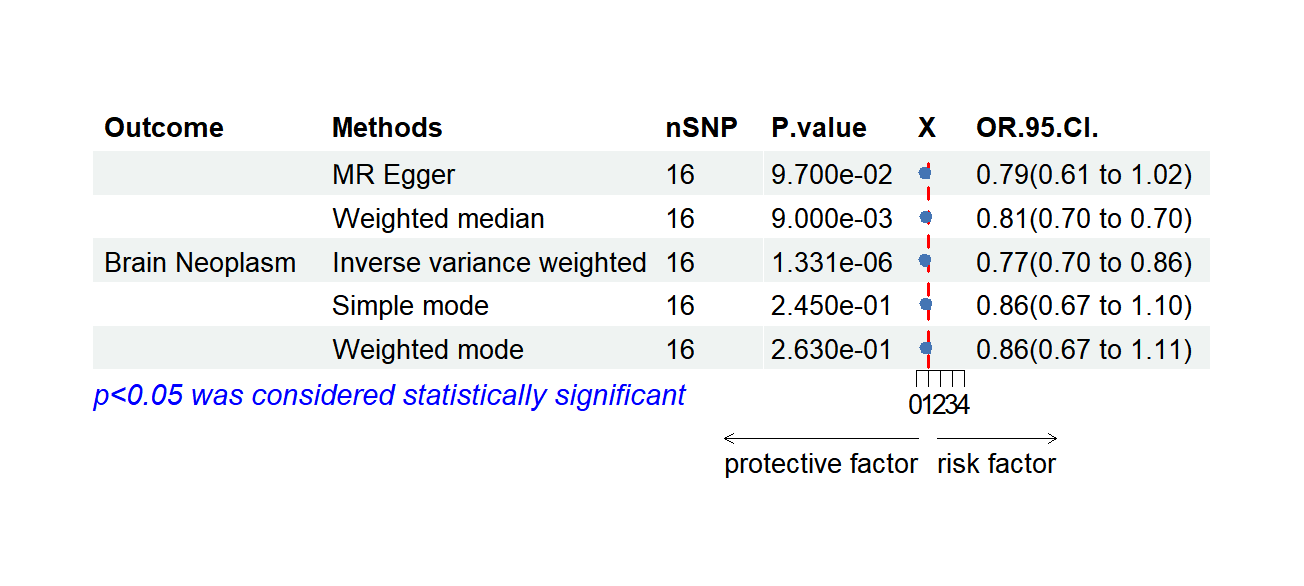

Supplement: Supplementary file 1 [file DataSheet1.zip › The data involved in the research - 2/Mendel Randomization/Rplot-4.png]

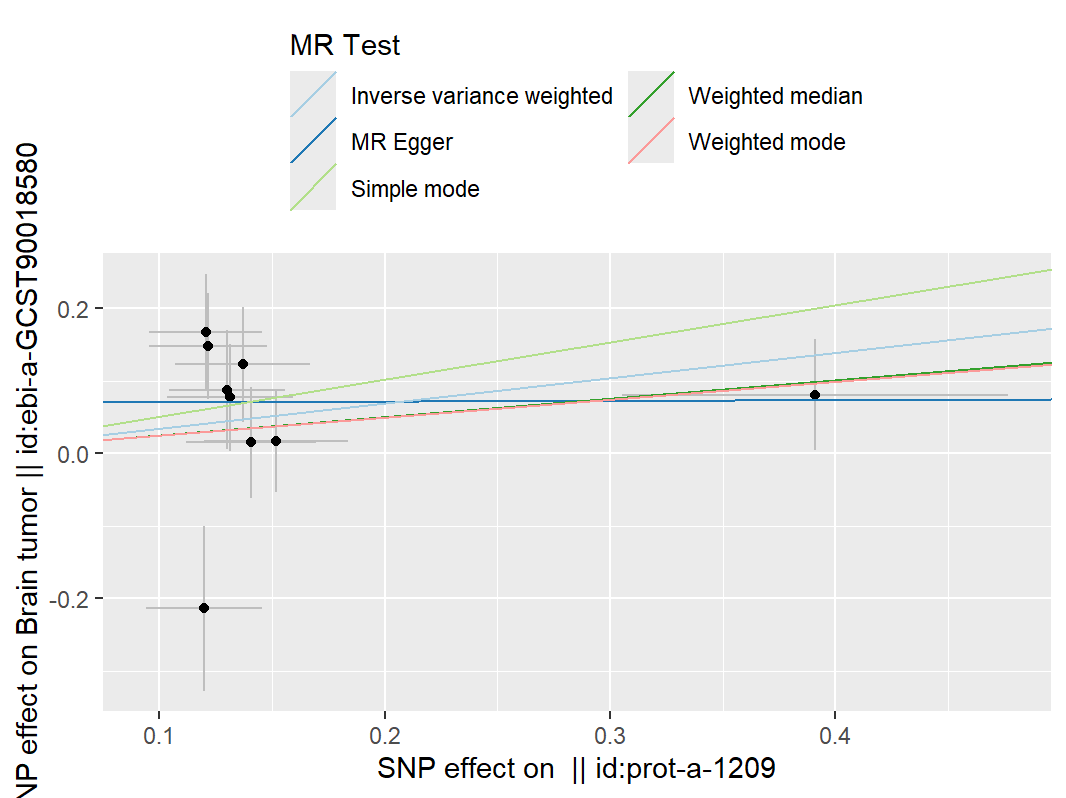

Supplement: Supplementary file 1 [file DataSheet1.zip › The data involved in the research - 2/Mendel Randomization/Scatter plots.png]

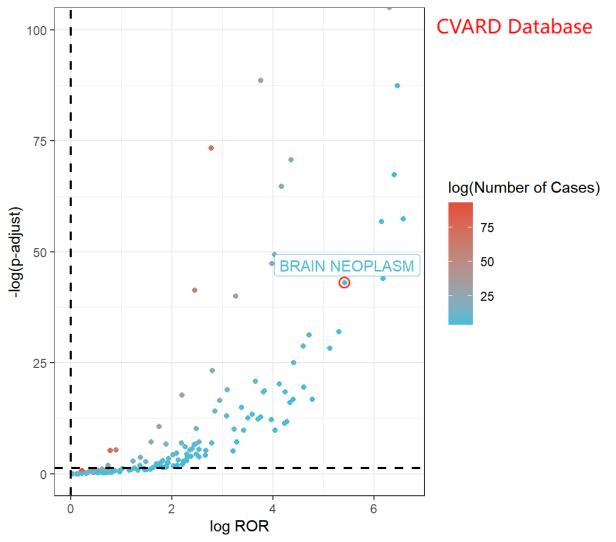

Supplement: Supplementary file 1 [file DataSheet1.zip › The data involved in the research - 2/Volcano map/CVARD.png]

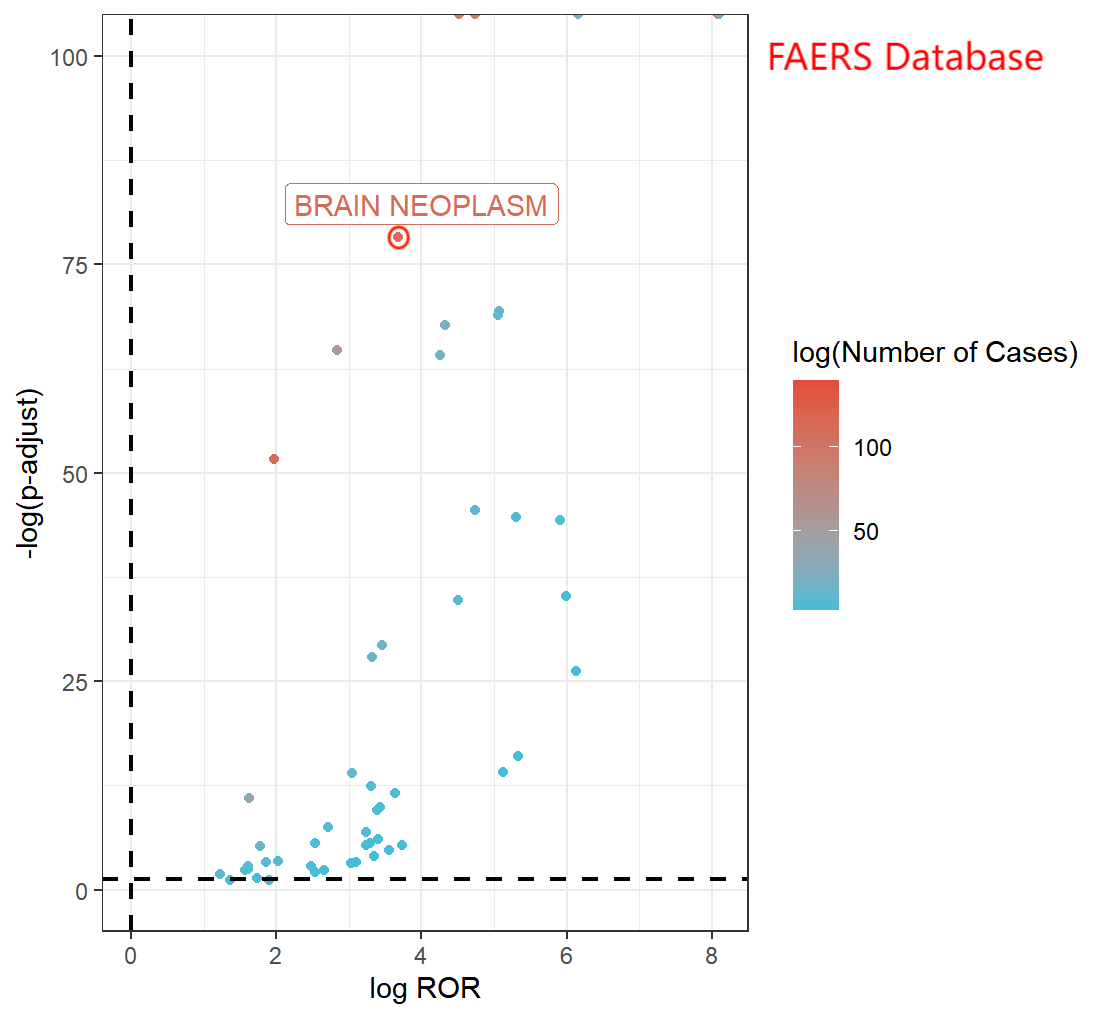

Supplement: Supplementary file 1 [file DataSheet1.zip › The data involved in the research - 2/Volcano map/FAERS.png]

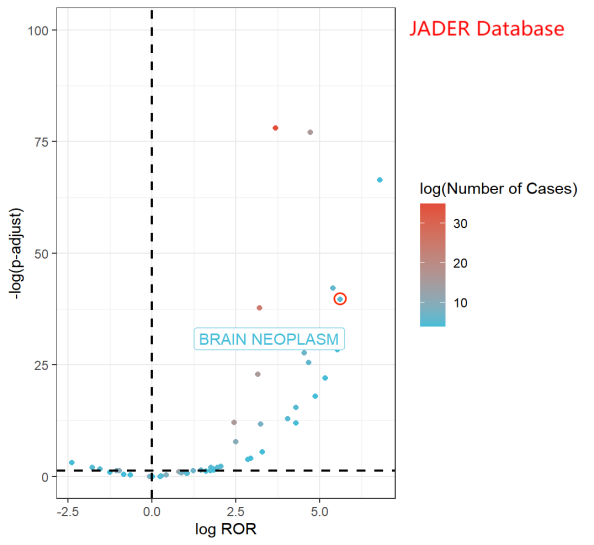

Supplement: Supplementary file 1 [file DataSheet1.zip › The data involved in the research - 2/Volcano map/JADER.png]
